# Dengue diagnostics: serious inaccuracies are likely to occur if pre-analytical conditions are not strictly followed

**DOI:** 10.1590/0074-02760200287

**Published:** 2021-01-29

**Authors:** Felipe Campos de Melo Iani, Ana Carolina Barbosa Caetano, Jéssica Caroline Wenceslau Cocovich, Frederico Figueiredo Amâncio, Maira Alves Pereira, Talita Émile Ribeiro Adelino, Sérgio Caldas, Marcos Vinícius Ferreira Silva, Glauco de Carvalho Pereira, Myrian Morato Duarte

**Affiliations:** 1 Fundação Ezequiel Dias, Diretoria do Instituto Octávio Magalhães, Serviço de Virologia e Riquetsioses, Belo Horizonte, MG, Brasil; 2 Centro Universitário de Belo Horizonte, Belo Horizonte, MG, Brasil; 3 Universidade Federal de Minas Gerais, Faculdade de Medicina, Programa de Pós-Graduação em Ciências da Saúde: Infectologia e Medicina Tropical, Belo Horizonte, MG, Brasil; 4 Fundação Ezequiel Dias, Diretoria de Pesquisa e Desenvolvimento, Serviço de Biotecnologia e Saúde, Belo Horizonte, MG, Brasil

**Keywords:** dengue, RNA, serum samples, storage conditions, diagnosis

## Abstract

**BACKGROUND:**

The heat-labile nature of Dengue virus (DENV) in serum samples must be considered when applying routine diagnostic tests to avoid issues that could impact the accuracy of test results with direct implications for case management and disease reporting.

**OBJECTIVES:**

To check if pre-analytical variables, such as storage time and temperature, have an impact on the accuracy of the main routine diagnostic tests for dengue.

**METHODS:**

Virus isolation, reverse transcription real-time polymerase chain reaction (RT-PCR) and NS1 enzyme-linked immunosorbent assay (ELISA) were evaluated using 84 samples submitted to different pre-analytical conditions.

**FINDINGS:**

Sensitivity and negative predictive value were directly affected by sample storage conditions. RT-PCR and virus isolation showed greater dependence on well-conserved samples for an accurate diagnosis. Interestingly, even storage at -30ºC for a relatively short time (15 days) was not adequate for accurate results using virus isolation and RT-PCR tests. On the other hand, NS1 ELISA showed no significant reduction in positivity for aliquots tested under the same conditions as in the previous tests.

**MAIN CONCLUSIONS:**

Our results support the stability of the NS1 marker in ELISA diagnosis and indicate that the accuracy of routine tests such as virus isolation and RT-PCR is significantly affected by inadequate transport and storage conditions of serum samples.

Dengue is a vector-borne viral disease transmitted to humans by the bite of female *Aedes* mosquitoes infected with Dengue virus (DENV). The spectrum of disease can range from asymptomatic or mild infection to severe complications, with plasma leakage leading to shock or fluid accumulation and breathing difficulties, in addition to severe organ involvement, such as the heart, among others.^1^ DENV belongs to the genus *Flavivirus*, family *Flaviviridae*, with four genetically related but antigenically distinct serotypes (DENV-1, -2, -3, and -4), each one including various subgroups called genotypes.^2^ Their positive single-stranded RNA genome (~10 kb) encodes three structural (envelope, E; membrane precursor, PrM; and capsid, C) and seven non-structural (NS1, NS2a, NS2b, NS3, NS4a, NS4b, and NS5) proteins, with important roles in replication, vector transmission, and virulence.^3^

After the onset of illness, DENV can be detected for 4-5 days in different specimens, such as blood samples (whole blood, serum, plasma) and other homogenised tissues (liver, lymph nodes, spleen, bone marrow) from fatal cases through virus isolation, reverse transcription real-time polymerase chain reaction (RT-PCR), or antigen detection.^4^ ^,^ ^5^ In some cases, the NS1 antigen has been detected for several days, even after defervescence, which is a critical period when infection may progress to severe dengue in some patients.^1^ Highly immunogenic NS1 is a conserved viral glycoprotein, expressed on the surface of infected cells and also secreted into the circulation, serving as a useful tool in early diagnosis of DENV infection since NS1 antigens remain in the blood for a long period of time.^6^ ^,^ ^7^ ^,^ ^8^ ^,^ ^9^ After the fifth day of symptoms, serology is the diagnostic method of choice, as antibodies gradually increase in the bloodstream, while viruses disappear.^1^ ^,^ ^6^ ^,^ ^10^ Although virus isolation is a time-consuming technique, in which viral viability and viral load are limiting parameters, cell culture infection is still considered the gold standard diagnostic procedure and is the most used method for DENV isolation and characterisation.^11^ ^,^ ^12^ *Aedes albopictus* C6/36 cells are some of the most commonly used cell lines for isolating DENV from serum samples.^13^ ^,^ ^14^ Since not all circulating DENV induces an easily identifiable cytopathic effect in C6/36 cells, infections of cell cultures must be confirmed by an immunofluorescence assay using serotype-specific monoclonal antibodies.^1^ ^,^ ^11^ ^,^ ^13^ However, molecular techniques such as RT-PCR are able to detect DENV within a considerably shorter time interval and with greater sensitivity than that achieved with the virus isolation technique.^11^ ^,^ ^15^

In most cases, DENV infection confers long-term immunity against the infecting serotype, but not against the other existing serotypes. In endemic areas, people may be infected several times throughout their lives, and secondary infection caused by a different serotype usually implies greater viremia and risk of developing severe disease.^11^ According to the World Health Organization,^16^ actual numbers of dengue cases are underreported and many of them are misclassified. Using cartographic approaches, Bhatt et al.^17^ found an estimated 390 million (95% credible interval 284-528 million) dengue infections per year worldwide, of which 96 million (67-136 million) manifest clinically (with any severity of disease).

The heat-labile nature of DENV, when isolated in serum, is an important factor directly related to laboratory diagnostic failures^1^ which may contribute, in addition to asymptomatic/oligosymptomatic cases not diagnosed or misclassified as other diseases, to the underestimation of the actual number of dengue cases. In Minas Gerais (MG), the Fundação Ezequiel Dias (Funed) is the Central Health Public Laboratory, responsible for the diagnosis of various diseases and disorders of compulsory notification. Despite Funed’s dengue diagnosis recommendations, MG health centres that collect patients’ sera usually do not have the appropriate facilities to maintain the samples until shipping. Thus, our main goal was to assess the association between adverse storage conditions of DENV-positive serum samples and the results generated by different diagnostic techniques routinely used by Funed (virus isolation, RT-PCR, and NS1 antigen-capture enzyme-linked immunosorbent assay - NS1 ELISA). Each of these tests has different purposes, and in many cases, they complement each other for a conclusive diagnosis. The NS1 ELISA is a low-cost and low-complexity test enabling the analysis of a large number of samples, which can be transported refrigerated, mainly for outbreaks and epidemics. On the other hand, RT-PCR and viral isolation are tests with greater complexity and a higher cost than the NS1 ELISA, being widely used to determine the serotype of the virus circulating in a given region. These tests demand greater care in the transport and storage of samples, which must be kept cryopreserved. In order to achieve this goal, we compared the accuracy of these three diagnostic tests in samples subjected to refrigeration or freezing as follows: (i) aliquots refrigerated for 24 h and 48 h compared to aliquots processed at time 0 h, i.e., immediately after thawing from liquid nitrogen, and (ii) aliquots frozen for 15 days compared to 0 h.

## MATERIALS AND METHODS

*Study design and population* - This was a cross-sectional experimental study in which 84 serum samples from patients with suspected dengue infection were analysed until the fourth day after the onset of symptoms. Samples were collected in the municipality of Betim/MG from March to May 2015, when high rates of dengue cases were being reported in the region.

*Ethics* - This study was approved by the Research Ethics Committee of the municipal health secretariat of Betim/Minas Gerais/Brazil under protocol number CAAE 44175415.5.0000.5651.

*Sample collection, storage, and transport* - Serum samples were obtained by centrifugation (10 min/2000 × g) of whole blood (10 mL) after collection, followed by immediate storage in liquid nitrogen. The samples were collected daily, and once a week the nitrogen container was transported to the Central Laboratory (Funed) in the state capital, Belo Horizonte, about 35 km from the city of Betim. As soon as the samples arrived in the laboratory, they were removed from the liquid nitrogen and divided into four aliquots, one of which was immediately tested (time 0 h) using the three methodologies described below. The aliquots were subsequently stored in a refrigerator (2-8ºC) for 24 h and 48 h, as well as in a freezer (-30ºC) for 15 days, in order to evaluate the impact of those variables, compared to time 0 h, on the accuracy of the routine tests used for samples collected during the viraemic period: virus isolation, RT-PCR, and NS1 ELISA.

*Virus isolation* - Samples were incubated with *Ae. albopictus* C6/36 cells cultured in L-15 medium (Leibovitz, GIBCO, USA) and supplemented with 2% foetal bovine serum (FBS). After 10 days of incubation at room temperature, mosquito cells were collected and viral isolates were checked using indirect immunofluorescence assay (IFA) according to Medina et al.^11^ IFA was performed with a monoclonal DENV antibody donated by Evandro Chagas Institute (Arbovirology and Haemorrhagic Fevers Section) and, according to Adungo et al.,^18^ a conjugated goat anti-mouse immunoglobulin G labelled with fluorescein isothiocyanate (FITC) (MP Biomedicals) with modifications. Images were obtained using an Olympus microscope, model BX51, with a DP72 camera and DP-2BSW software.

*Nucleic acid isolation and reverse transcription real-time PCR* - Viral RNA was extracted using the QIAamp Viral RNA Mini Kit (Qiagen) starting with 140 µL of serum and following the manufacturer’s recommendations. The real-time PCR was performed using the QuantiTect Probe RT-PCR Kit (Qiagen) in a 25 µL reaction with primers and probes from the Centers for Disease Control and Prevention’s (CDC) DENV-1-4 Real-Time RT-PCR (CDC, Atlanta, USA)^19^ according to the manufacturer’s recommendations. The RT-PCR was carried out in a 7500 Real-Time PCR System (Applied Biosystems), and the cycling profile of this assay consisted of a 15 min RT step that was performed at 50ºC and 2 min of Taq polymerase activation at 95ºC, followed by 45 cycles of PCR at 95ºC, denaturing for 15 s and annealing/extension at 60ºC for 1 min.

*NS1 antigen-capture ELISA* - Detection of the NS1 protein was performed by ELISA using the Platelia™ dengue NS1 Ag kit (Bio-Rad) according to the manufacturer’s recommendations with 50 µL serum samples.

*Statistical analyses* - Data from aliquots stored in a refrigerator (2-8ºC) for 24 h and 48 h and in a freezer (-30ºC) for 15 days were compared to those from aliquots processed immediately after thawing from liquid nitrogen (0 h), considering the reference condition. The McNemar test was used to check the agreement between different sample storage conditions and time 0 h for each technique using GraphPad (https://graphpad.com/quickcalcs/mcNemar1/). The OpenEpi Version 3 calculator was used to estimate the sensitivity, specificity, positive/negative predictive value, diagnostic accuracy, and Cohen’s kappa value for samples stored under different conditions compared to time 0 h.

## RESULTS

In this study, 84 serum samples were used to verify the impact of refrigerator and freezer pre-storage on the performance of three dengue diagnostic tests provided by Funed (virus isolation, RT-PCR, and NS1 ELISA).

**Fig. 1:**
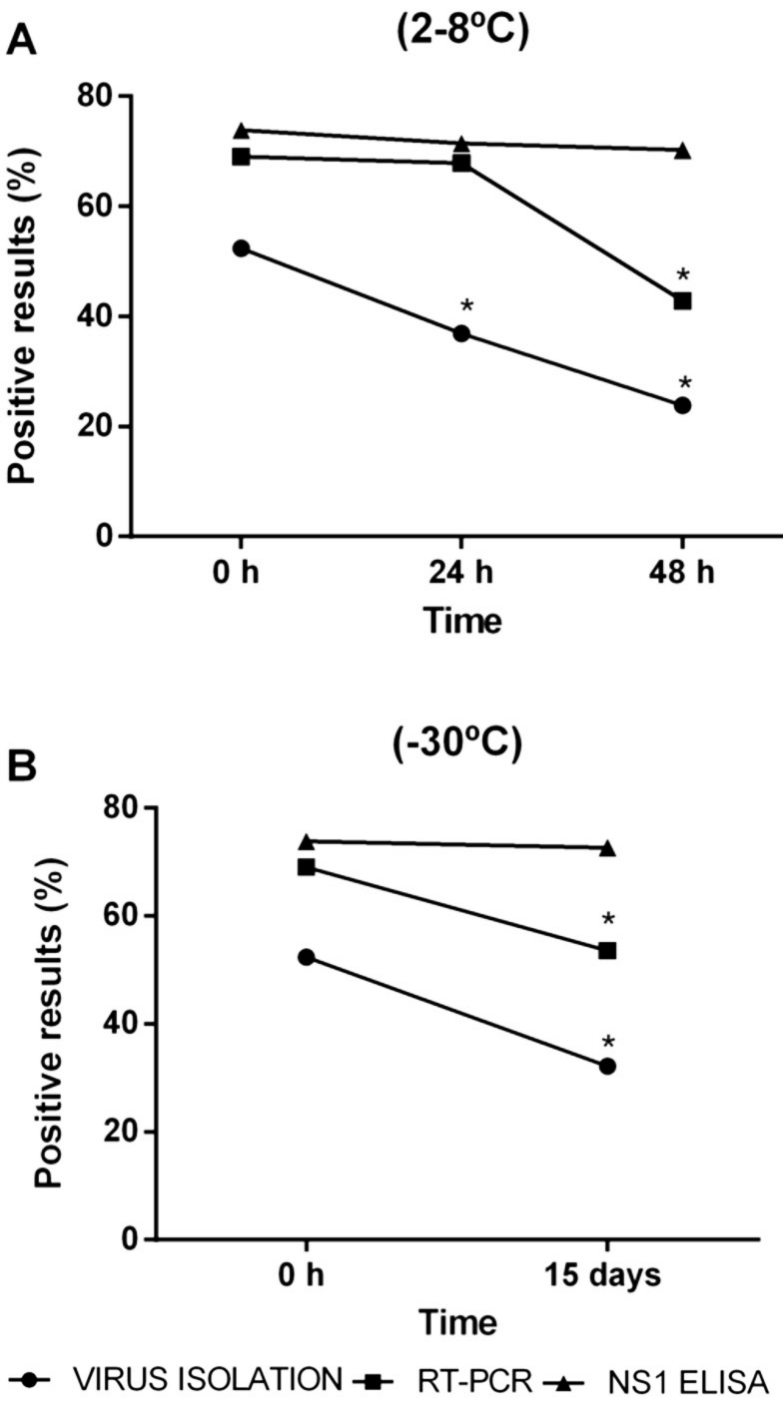
percentage of samples testing positive for dengue by virus isolation, real-time polymerase chain reaction (RT-PCR), and NS1 enzyme-linked immunosorbent assay (ELISA) compared to analysis performed immediately after thawing from liquid nitrogen and fractionation (time 0 h). (A) Analysis performed with aliquots refrigerated for 24 h and 48 h at 2-8ºC. (B) Analysis performed with aliquots frozen for 15 days at -30ºC. The asterisk denotes a significant difference relative to time 0 h for each test (p < 0.5, McNemar test).

**Fig. 2:**
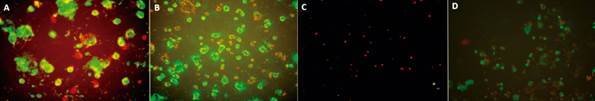
typical indirect immunofluorescence reaction after the virus isolation procedure. (A) Positive sample at 0 h and 40× magnification. (B) Positive sample at 24 h and 40× magnification. (C) Negative sample at 48 h and 10× magnification. (D) Positive sample at 15 days and 40× magnification. Dengue virus-positive cells fluoresce by binding to a primary anti-dengue monoclonal IgG antibody followed by a fluorescein isothiocyanate (FITC)-conjugated secondary anti-IgG antibody.

As can be seen in Fig. 1A, which presents data from samples stored in a refrigerator, NS1 ELISA showed the highest detection of positive results (73.8%) for suspected DENV samples, and no significant variation in positivity was observed when the storage time increased. In a similar way, RT-PCR also showed a high percentage of positive results for aliquots processed immediately after fractionation (69.0%) or after up to 24 h of storage (67.9%). However, after 48 h in a refrigerator, the positivity decreased significantly to 42.9%. On the other hand, of the three tests, virus isolation showed the lowest detection of positive results at time 0 h (52.4%), presenting a significant reduction in positivity at storage times of 24 h (36.9%) and 48 h (23.8%). For frozen samples (Fig. 1B), NS1 ELISA again showed great consistency in assay performance, as observed in the refrigerated sera, showing no significant reduction in the percentage of positive results (72.6%) when compared to time 0 h (73.8%). However, RT-PCR and virus isolation techniques showed a reduction in positivity to 53.6% and 32.1% of the samples, respectively. Fig. 2 shows typical reactive and nonreactive results of the indirect immunofluorescence technique performed after the virus isolation assay. Although performed for non-quantitative purposes, it is noteworthy that with increasing time in the refrigerator, as well as when frozen at -30ºC for 15 days, some samples showed a clear decline in fluorescence compared to time 0 h.

Considering time 0 h as the reference standard for each diagnostic test, the loss of positivity observed respectively at 24 h (2-8ºC), 48 h (2-8ºC), and 15 days (-30ºC) were 15.5%, 28.6%, and 20.2% for virus isolation; 1.2%, 26.2%, and 15.5% for RT-PCR; and 2.4%, 3.6%, and 1.2% for NS1 ELISA (Table I).

**TABLE I:** Loss of positivity compared to results observed at time 0 h, which was considered the reference standard for the analysis of time and temperature variations for each diagnostic test

	Loss of positivity in relation to time 0 h/ N = 84 (%)		
Time (Temperature)	Virus isolation	RT-PCR	NS1 ELISA
24 h (2-8**º**C)	13 (15.5)	1 (1.2)	2 (2.4)
48 h (2-8ºC)	24 (28.6)	22 (26.2)	3 (3.6)
15 days (-30ºC)	17 (20.2)	13 (15.5)	1 (1.2)

ELISA: enzyme-linked immunosorbent assay; RT-PCR: real-time polymerase chain reaction.

Finally, compared to time 0 h, the performance evaluation of each diagnostic test using samples refrigerated for 24 h and 48 h, as well as frozen for 15 days, demonstrated respectively that sensitivity (sens), negative predictive value (npv), diagnostic accuracy (acc), and Cohen’s Kappa (κ) were the lowest for virus isolation (sens = 70.5%, 45.5%, and 61.4%; npv = 75.5%, 62.5%, and 70.2%; acc = 84.5%, 71.4%, and 79.8%; κ = 0.7, 0.4, and 0.6) when compared to RT-PCR (sens = 98.3%, 62.1%, and 77.6%; npv = 96.3%, 54.2%, and 66.8%; acc = 98.8%, 73.8%, and 84.5%; κ = 1.0, 0.5, and 0.7) and NS1 ELISA (sens = 96.8%, 95.2%, and 98.4%; npv = 91.7%, 88%, and 95.6%; acc = 97.6%, 96.4%, and 98.8%; κ = 0.9, 0.9, and 1.0) (Table II). Raw data from the 84 serum samples used in this study can be seen in the Supplementary data (Table).

**TABLE II:** Performance parameters of virus isolation, real-time polymerase chain reaction (RT-PCR), and NS1 enzyme-linked immunosorbent assay (ELISA) diagnostic tests performed with refrigerated samples (2-8ºC for 24 h and 48 h) and frozen samples (-30ºC for 15 days) compared to time 0 h

		24 h (2-8**º**C)		48 h (2-8**º**C)		15 days (-30**º**C)	
Parameter		Value	CI 95%^*a*^	Value	CI 95%^*a*^	Value	CI 95%^*a*^
Virus isolation	Sens	70.5%	(55.8, 81.8^*b*^ )	45.5%	(31.7, 59.9^*b*^ )	61.4%	(46.6, 74.3^*b*^ )
	Spec	100%	(91.2, 100^*b*^ )	100%	(91.2, 100^*b*^ )	100%	(91.2, 100^*b*^ )
	PPV	100%	(89.0, 100^*b*^ )	100%	(83.9, 100^*b*^ )	100%	(87.5, 100^*b*^ )
	NPV	75.5%	(62.4, 85.1^*b*^ )	62.5%	(50.3, 73.3^*b*^ )	70.2%	(57.3, 80.5^*b*^ )
	Acc	84.5%	(75.3, 90.7^*b*^ )	71.4%	(61.0, 80.0^*b*^ )	79.8%	(70.0, 87.0^*b*^ )
	Kappa	0.7	(0.5 - 0.9)	0.4	(0.3 - 0.6)	0.6	(0.4 - 0.8)
RT-PCR	Sens	98.3%	(90.9, 99.7^*b*^ )	62.1%	(49.2, 73.4^*b*^ )	77.6%	(65.3, 86.4^*b*^ )
	Spec	100%	(87.1, 100^*b*^ )	100%	(87.1, 100^*b*^ )	100%	(87.1, 100^*b*^ )
	PPV	100%	(93.7, 100^*b*^ )	100%	(90.4, 100^*b*^ )	100%	(92.1, 100^*b*^ )
	NPV	96.3%	(81.7, 99.3^*b*^ )	54.2%	(40.3, 67.4^*b*^ )	66.8%	(51.0, 79.4^*b*^ )
	Acc	98.8%	(93.6, 99.8^*b*^ )	73.8%	(63.5, 82.0^*b*^ )	84.5%	(75.3, 90.7^*b*^ )
	Kappa	1.0	(0.8 - 1.2)	0.5	(0.3 - 0.7)	0.7	(0.5 - 0.9)
NS1 ELISA	Sens	96.8%	(89.0, 99.1^*b*^ )	95.2%	(86.7, 98.3^*b*^ )	98.4%	(91.4, 99.7^*b*^ )
	Spec	100%	(85.1, 100^*b*^ )	100%	(85.1, 100^*b*^ )	100%	(85.1, 100^*b*^ )
	PPV	100%	(94.0, 100^*b*^ )	100%	(94.0, 100^*b*^ )	100%	(94.1, 100^*b*^ )
	NPV	91.7%	(74.1, 97.7^*b*^ )	88%	(70.0, 95.6^*b*^ )	95.6%	(79.0, 99.2^*b*^ )
	Acc	97.6%	(91.7, 99.3^*b*^ )	96.4%	(90.0, 98.8^*b*^ )	98.8%	(93.6, 99.8^*b*^ )
	Kappa	0.9	(0.7 - 1.2)	0.9	(0.7 - 1.1)	1.0	(0.8 - 1.2)

*a*: confidence interval 95% lower-higher; *b*: Wilson score method; Sens: sensitivity; Spec: specificity; PPV: positive predictive value; NPV: negative predictive value; Acc: diagnostic accuracy; Kappa: Cohen’s Kappa (Unweighted).

## DISCUSSION

An accurate diagnosis is essential for epidemiology and the health of infected individuals. DENV infection produces a wide spectrum of symptoms, and many of them are not specific. Therefore, a diagnosis based only on clinical symptoms is not reliable, and the correct clinical management of the patient depends on an early and accurate diagnosis.^20^ In addition, early laboratory confirmation of clinical diagnosis can be life-saving since some patients progress from mild to severe disease, and sometimes even death, in a short period of time.^1^ ^,^ ^20^

Species-specific identification using techniques such as virus isolation, confirmed by immunofluorescence assay,^11^ as well as RT-PCR, are strategies used by reference laboratories for differential and accurate diagnosis. Nevertheless, the differentiation between dengue and other similar diseases (like leptospirosis, Zika, chikungunya, and others) is essential for correct patient prognosis and effective epidemiological surveillance.^12^ ^,^ ^21^ Another important aspect for epidemiological surveillance, as well as for guiding government actions to combat the disease, is reliable reporting of dengue cases. However, such notifications are often underestimated. Diagnostic failures generated by improper handling of samples during collection, transport, and storage may contribute to this scenario. In addition, the occurrence of mild and/or asymptomatic clinical manifestations may contribute to the underreporting of dengue cases since many individuals under these conditions do not seek medical attention.^16^ ^,^ ^17^ In this study, we did not observe a decrease in specificity or positive predictive value for any of the conditions and diagnostic tests evaluated. Specificity is defined as the proportion of truly nondiseased people who have tested negative using a diagnostic test, and positive predictive value is the proportion of people with a positive test result who truly have the disease.^22^ ^,^ ^23^ However, we observed that the negative predictive value, defined as the probability of a negative test accurately predicting disease absence,^22^ ^,^ ^23^ was directly affected by the different conditions in which the samples were stored. Here, NS1 ELISA showed the highest negative predictive values, for samples refrigerated for 48 h (88%) or frozen for 15 days (95.6%), being slightly lower (91.7%) than RT-PCR (96.3%) in relation to samples refrigerated for 24 h. However, RT-PCR showed a decrease in this proportion to 66.8% and 54.2% for samples stored at -30ºC for 15 days and refrigerated at 2-8ºC for 48 h, respectively. In addition, virus isolation showed the lowest negative predictive value for samples refrigerated 24 h (75.5%) and intermediate proportions compared to the other two tests for samples refrigerated for 48 h (62.5%) and frozen for 15 days (70.2%). Thus, compared to time 0 h, we observed a loss of positivity in about 26-28% of the aliquots analysed using both virus isolation and RT-PCR after 48 h of refrigerator storage. Although RT-PCR showed no significant diagnostic failure for aliquots processed after 24 h at 2-8ºC, those subjected to virus isolation at this same time were highly susceptible to loss of viability, demonstrating an increase of negative results in 15.5% of the samples. Interestingly, even storage at -30ºC for a relatively short period of time (15 days) was not adequate for virus isolation and RT-PCR tests, as additional reductions in positivity were seen in about 20% and 15% of these tests, respectively. Such diagnostic failures are likely to occur because virus isolation depends on viral viability, which, as our data emphasise, is highly sensitive to conditions of transport, storage, and handling. It is important to note that virus isolation proved to be the technique most harmed by sample pre-processing conditions since at time 0 h, virus isolation detected 52.4% of positive samples, while RT-PCR and NS1 ELISA detected 69.0% and 73.8%, respectively (Fig. 1). Compared to time 0 h, virus isolation showed a decrease in its diagnostic sensitivity to 70.5% at 24 h and 45.5% at 48 h for refrigerated samples, as well as to 61.4% for samples cryopreserved at -30ºC for 15 days. Although the immunofluorescence test used after virus isolation was non-quantitative, we noticed a clear reduction in fluorescence in many samples and, consequently, in the number of infected cells. It is reasonable to speculate that samples with very low virus levels may present false-negative results, especially in the virus isolation assay. In addition, the viral adsorption required for DENV isolation is likely to be compromised due to the instability of heat-sensitive surface proteins involved in the cell interaction process.^24^

Nevertheless, RNA is more unstable than DNA and susceptible to the action of RNAses when not properly stored, being a possible reason for the decrease in RT-PCR accuracy, directly influenced by the pre-analytical conditions assessed.

In fact, immunofluorescence assays are not as sensitive as other DENV detection protocols, such as the CDC real-time RT-PCR assay used here, which, in addition, has a much faster turnaround time for analysis.^1^ ^,^ ^11^ ^,^ ^25^ Sensitivity is defined as the proportion of truly sick people who have tested positive in a diagnostic test.^22^ ^,^ ^23^ In general, when correctly performed, PCR has several advantages as a diagnostic method, such as high sensitivity and high throughput sample processing in relation to virus isolation and serotyping. PCR has been used in samples stored for long periods;^21^ however, as seen in our findings, even this technique may have reduced sensitivity if samples were not stored at appropriate temperatures and time conditions. The sensitivity of RT-PCR for samples refrigerated for 24 h was 98.3%, dropping to 62.1% after 48 h, and also decreasing when samples were cryopreserved for 15 days (77.6%). An applicable alternative for cost efficiency and convenience, maintaining the integrity of viral RNA, would be the use of lysis/binding buffer to store and transport serum to the diagnostic laboratory, where RNA extraction could be performed by silica-based adsorption methods, without the need for cold-chain storage.^26^ However, viral isolation necessarily requires the preservation of DENV viability, making storage below -70ºC recommended for the accuracy of the assay.^26^ ^,^ ^27^ Another hurdle for the implementation of such alternative diagnostic testing outside the Central Laboratory had to do with the lack of adequately trained staff and diagnostic infrastructure. On the other hand, DENV NS1 seemed to remain stable for longer than the viral genome when in solution, in addition to being secreted at high levels during viral infection with significant accumulation in the serum of infected individuals.^9^ ^,^ ^28^ Experimentally, the NS1 protein was observed to be predominantly associated with the liver, demonstrating efficient internalisation and marked stability in human hepatocytes.^7^ According to Alcon-LePoder et al.,^7^ the possibility that NS1 may contribute to viral propagation in vivo is evidenced by the observation of NS1 accumulation in the late endosomal compartment of hepatocytes and the subsequent potentiation of DENV infection in vitro. Using a transgenic expression system, it has been shown that the DENV NS1 glycan N130 is required for stabilisation of the secreted NS1 hexamer.^8^ ^,^ ^9^ Our data point to NS1 ELISA as the most sensitive and stable diagnosis technique for DENV infection since its diagnostic performance parameters remained constant at all times and temperatures analysed. According to Altman,^23^ the strength of agreement for interpreting kappa values can be classified as follows: “poor” (< 0.2), “fair” (0.2-0.4), “moderate” (0.4-0.6), “good” (0.6-0.8), and “very good” (0.8-1.0). Consistently, the interpretation of the kappa value (considering time 0 h as the reference standard for each test) showed a “very good” agreement of the results for NS1 ELISA (κ ≥ 0.9 in all analyses) as well as for RT-PCR with samples refrigerated for 24 h (κ = 1.0). However, for samples refrigerated for 48 h, the agreements were classified as “moderate” for virus isolation (κ = 0.4) and RT-PCR (κ = 0.5), and as “good agreement” (0.6 < κ < 0.8) for the other conditions and tests.^23^ ^,^ ^29^

However, a disadvantage of NS1 ELISA is that the viral serotype is not provided by detection of the NS1 protein,^12^ ^,^ ^25^ and the viral serotype is important for clinical and epidemiological studies since the introduction of a new serotype in a given area may alter the dynamics of dengue in the affected population.^21^ In addition, the cross reactivity of human anti-NS1 antibodies appears to increase after sequential infections of DENV and Zika virus.^30^ Nevertheless, the use of different routine diagnostic tests in the fields of virology, molecular biology, and serology is a valuable strategy to circumvent the limitations of a single isolated technique, mainly for the resolution of inconclusive cases influenced by variations in diagnostic accuracy. In our study, the diagnostic accuracy was greater than 96% for all NS1 ELISA analyses, with similar results for RT-PCR using samples refrigerated for 24 h. The lowest diagnostic accuracy under this same condition of analysis was observed using virus isolation (84.5%).

Regarding the tests performed with samples refrigerated for 48 h and frozen for 15 days, the diagnostic accuracy for virus isolation and RT-PCR was similarly compromised, with accuracies around 71-74% (cooling for 48 h) and 79-85% (freezing for 15 days).

Therefore, this study supports the procedure established by Funed to standardise the pre-analytical conditions for transporting and storing samples in all MG health centres in order to ensure diagnostic accuracy. Thus, containers with liquid nitrogen and freezing cryotubes have been periodically sent to MG public health centres, and health professionals involved in collection, pre-processing, and transportation have been properly trained.

Although we did not evaluate cross-reactivity with Zika virus, our data demonstrated the robustness of NS1 antigen detection ELISA for dengue diagnosis, since there was no loss of accuracy resulting from pre-analytical conditions, such as storage in a refrigerator for 24 h or 48 h and/or a freezer for 15 days. Finally, the accuracy of the other two routine tests, virus isolation and RT-PCR, was seriously affected by variations in such pre-analytical conditions. Thus, based on our data and routine experience, we advise laboratories to store samples in liquid nitrogen, both in collection centres and during transport. In addition, we recommend storing the serum in a -80ºC freezer if it is not possible to process the samples immediately after removal from liquid nitrogen.
