# Supplementary material for: Dengue diagnostics: serious inaccuracies are likely to occur if pre-analytical conditions are not strictly followed
Source: Mem Inst Oswaldo Cruz. 2021 Jan 29;115:e200287. doi: 10.1590/0074-02760200287 (PMC7849175; doi:10.1590/0074-02760200287)
Supplement: Supplementary file 1 [file 1678-8060-mioc-115-e200287-s.pdf]

TABLE

Raw data from serum samples submitted to different dengue diagnostic methods after refrigeration (2-8ºC) for 24 h and 48 h, and after freezing (–30ºC) for 15 days, compared to the respective fractionated aliquots tested immediately after thawing from liquid nitrogen (time 0 h)

	Virus isolation#				NS1 ELISA				RT-PCR			
SN	0 h	24 h	48 h	15 days	0 h	24 h	48 h	15 days	0 h	24 h	48 h	15 days
1	D1	D1	N	D1	RE	RE	RE	RE	D1	D1	D1	D1
2	N	N	N	N	NR	NR	NR	NR	ND	ND	ND	ND
3	D1	D1	N	D1	RE	RE	RE	RE	D1	D1	D1	D1
4	D1	D1	D1	D1	RE	RE	RE	RE	D1	D1	D1	D1
5	N	N	N	N	RE	RE	ID*	RE	D1	D1	D1	D1
6	N	N	N	N	NR	NR	NR	NR	ND	ND	ND	ND
7	D1	D1	D1	N	RE	ID*	NR	RE	D1	D1	D1	D1
8	D1	D1	D1	D1	RE	RE	RE	RE	D1	D1	D1	D1
9	D1	D1	N	D1	RE	RE	RE	RE	D1	D1	D1	D1
10	N	N	N	N	NR	NR	NR	NR	D1	ND	ND	ND
11	D1	D1	N	D1	RE	RE	RE	RE	D1	D1	D1	D1
12	D1	D1	N	D1	RE	RE	RE	RE	D1	D1	D1	D1
13	D1	N	N	N	RE	RE	RE	RE	D1	D1	ND	D1
14	N	N	N	N	NR	NR	NR	NR	ND	ND	ND	ND
15	N	N	N	N	RE	RE	RE	RE	D1	D1	D1	D1
16	N	N	N	N	NR	NR	NR	NR	ND	ND	ND	ND
17	D1	D1	D1	D1	RE	RE	RE	RE	D1	D1	ND	D1
18	N	N	N	N	RE	RE	RE	RE	D1	D1	ND	ND
19	N	N	N	N	NR	NR	NR	NR	ND	ND	ND	ND
20	N	N	N	N	NR	NR	NR	NR	ND	ND	ND	ND
21	N	N	N	N	NR	NR	NR	NR	ND	ND	ND	ND
22	D1	N	N	N	RE	RE	RE	RE	D1	D1	D1	D1
23	N	N	N	N	NR	NR	NR	NR	ND	ND	ND	ND
24	D1	N	N	N	RE	RE	RE	RE	D1	D1	ND	ND
25	N	N	N	N	RE	RE	RE	RE	D1	D1	ND	D1
26	D1	N	N	D1	RE	RE	RE	RE	D1	D1	D1	D1
27	D1	D1	D1	D1	RE	RE	RE	RE	D1	D1	D1	D1
28	N	N	N	N	RE	RE	RE	RE	D1	D1	D1	D1
29	D1	D1	N	D1	RE	RE	RE	RE	D1	D1	D1	D1
30	N	N	N	N	NR	NR	NR	NR	D1	D1	D1	D1
31	N	N	N	N	NR	NR	NR	NR	ND	ND	ND	ND
32	D1	D1	N	D1	RE	RE	RE	RE	D1	D1	D1	D1
33	N	N	N	N	NR	NR	NR	NR	ND	ND	ND	ND
34	N	N	N	N	RE	RE	RE	RE	D1	D1	ND	D1
35	N	N	N	N	RE	RE	RE	RE	D1	D1	ND	ND
36	D1	D1	D1	D1	RE	RE	RE	RE	D1	D1	D1	ND
37	D1	D1	N	D1	RE	RE	RE	RE	D1	D1	D1	D1
38	N	N	N	N	RE	RE	RE	RE	D1	D1	ND	ND
39	D1	D1	D1	D1	RE	RE	RE	RE	D1	D1	D1	D1
40	D1	N	N	D1	RE	RE	RE	RE	D1	D1	D1	D1
41	D1	N	N	N	RE	RE	RE	RE	D1	D1	D1	D1
42	D1	D1	N	N	RE	RE	RE	RE	D1	D1	D1	ND
43	N	N	N	N	NR	NR	NR	NR	ND	ND	ND	ND

CC BY

	Virus isolation#				NS1 ELISA				RT-PCR			
SN	0 h	24 h	48 h	15 days	0 h	24 h	48 h	15 days	0 h	24 h	48 h	15 days
44	N	N	N	N	NR	NR	NR	NR	ND	ND	ND	ND
45	N	N	N	N	NR	NR	NR	NR	ND	ND	ND	ND
46	N	N	N	N	RE	RE	RE	RE	D1	D1	ND	D1
47	N	N	N	N	RE	RE	RE	RE	ND	ND	ND	ND
48	D1	N	N	N	RE	RE	RE	RE	D1	D1	ND	ND
49	D1	D1	D1	D1	RE	RE	RE	RE	D1	D1	D1	D1
50	D1	D1	D1	D1	RE	RE	RE	RE	D1	D1	D1	D1
51	D1	D1	D1	N	RE	RE	RE	RE	D1	D1	D1	D1
52	N	N	N	N	NR	NR	NR	NR	ND	ND	ND	ND
53	D1	D1	D1	N	RE	RE	RE	RE	D1	D1	D1	D1
54	N	N	N	N	NR	NR	NR	NR	ND	ND	ND	ND
55	N	N	N	N	NR	NR	ID*	NR	ND	ND	ND	ND
56	N	N	N	N	RE	RE	RE	RE	D1	D1	ND	ND
57	N	N	N	N	NR	NR	NR	NR	ND	ND	ND	ND
58	N	N	N	N	RE	RE	RE	RE	ND	ND	ND	ND
59	N	N	N	N	RE	RE	RE	RE	ND	ND	ND	ND
60	D1	N	N	N	RE	RE	RE	RE	D1	D1	ND	D1
61	D1	D1	D1	N	RE	RE	RE	RE	D1	D1	D1	D1
62	D1	D1	D1	D1	RE	RE	RE	RE	D1	D1	D1	D1
63	N	N	N	N	RE	RE	RE	RE	ND	ND	ND	ND
64	D1	D1	D1	D1	RE	RE	RE	RE	D1	D1	D1	D1
65	D1	N	N	N	RE	RE	RE	RE	D1	D1	ND	ND
66	D1	D1	D1	D1	RE	RE	RE	RE	D1	D1	D1	D1
67	N	N	N	N	RE	RE	RE	RE	D1	D1	ND	D1
68	D1	D1	D1	D1	RE	RE	RE	RE	D1	D1	D1	D1
69	D1	D1	D1	D1	RE	RE	RE	RE	D1	D1	D1	D1
70	N	N	N	N	NR	NR	NR	NR	ND	ND	ND	ND
71	N	N	N	N	ID*	NR	NR	NR	ND	ND	ND	ND
72	D1	N	N	N	RE	RE	RE	RE	D1	D1	ND	ND
73	D1	D1	N	D1	RE	RE	RE	RE	D1	D1	ND	D1
74	D1	D1	D1	D1	RE	RE	RE	RE	D1	D1	D1	D1
75	N	N	N	N	NR	NR	NR	ID*	ND	ND	ND	ND
76	D1	D1	N	N	RE	RE	RE	RE	D1	D1	ND	D1
77	D1	N	N	N	RE	RE	RE	RE	D1	D1	ND	D1
78	D1	D1	D1	D1	RE	RE	RE	RE	D1	D1	D1	D1
79	D1	N	N	N	RE	RE	RE	RE	D1	D1	ND	D1
80	N	N	N	N	RE	NR	NR	NR	ND	ND	ND	ND
81	D1	D1	D1	D1	RE	RE	RE	RE	D1	D1	ND	D1
82	N	N	N	N	RE	RE	RE	RE	D1	D1	ND	ND
83	N	N	N	N	RE	RE	RE	RE	ND	ND	ND	ND
84	D1	N	N	N	RE	RE	RE	RE	D1	D1	D1	ND

SN: sample number; D1: Dengue virus 1; N: negative; RE: reactive; NR: nonreactive; ID: indeterminate; ND: not detected; #: virus isolation was confirmed by an immunofluorescence assay;[11] *: samples with indeterminate results in NS1 ELISA were analysed as NR: samples 5, 7, 55, 71, and 75.
